# A Case of Chronic Expanding Hematoma in the Thoracic Cavity Treated with Transcatheter Arterial Embolization

**DOI:** 10.3400/avd.cr.25-00148

**Published:** 2026-03-18

**Authors:** Takahiro Higuchi, Shunsuke Inaki, Akira Baba, Keitaro Enoki, Takayuki Suzuki, Hideomi Yamauchi, Tetsuya Shimizu, Saeko Kubomae, Haruki Mori, Naoki Kurata

**Affiliations:** 1 Department of Radiology, The Jikei University School of Medicine, Kashiwa Hospital, Kashiwa, Chiba, Japan; 2 Department of Pulmonary Medicine, The Jikei University School of Medicine, Kashiwa Hospital, Kashiwa, Chiba, Japan

**Keywords:** hematoma, chronic expanding hematoma, thoracic cavity, transcatheter arterial embolization

## Abstract

Chronic expanding hematoma (CEH) is a rare late complication of thoracic trauma or surgery, and favorable outcomes with transcatheter arterial embolization (TAE) have rarely been reported. An 80-year-old male presented with recurrent hemoptysis and was diagnosed with intrathoracic CEH. Owing to advanced age and high surgical risk, TAE was performed via bronchial, intercostal, and inferior phrenic arteries using tris-acryl microspheres (700–900 μm) and gelatin sponge. Hemoptysis resolved, and follow-up imaging showed hematoma shrinkage without recurrence for 2 years. This case demonstrates TAE with 700–900 μm microspheres as a safe, minimally invasive alternative to surgery for CEH.

## Introduction

Chronic expanding hematoma (CEH) is a rare condition that develops in various soft tissues of the body, involving a progressively enlarging hematoma that typically occurs long after an initial triggering event, such as trauma, surgery, or infection.^1)^ In the thoracic cavity, CEH commonly occurs as a late complication of surgery or chest trauma.

CEH may present asymptomatically or with symptoms such as hemoptysis, dyspnea, or hoarseness.^2)^ Management strategies range from surgical resection to minimally invasive options such as transcatheter arterial embolization (TAE), depending on the overall health of the patient and the lesion’s size and location. Although surgical excision is the first choice for curative treatment, TAE may offer a minimally invasive alternative. Previous reports on the use of TAE alone for the treatment of CEH are limited, although the outcomes have generally been unfavorable.^3)^ Herein, we present a case of intrathoracic CEH treated solely with TAE, resulting in a favorable clinical course.

## Case Report

An 80-year-old male presented with hemoptysis. Imaging examinations conducted at another hospital revealed a mass in the right lower lung field of unclear etiology. The patient had experienced mild hemoptysis 8 months prior, followed by recurrent hemoptysis and cough 1 month before presenting to our hospital.

His past medical history included a right artificial pneumothorax for pulmonary tuberculosis (unknown timing) and cerebral infarction 2 years before presentation. His medication included aspirin, and he had a smoking history of 5–6 cigarettes per day, although he had quit smoking at the age of 50 years.

### Imaging and clinical findings

**Fig. 1:**
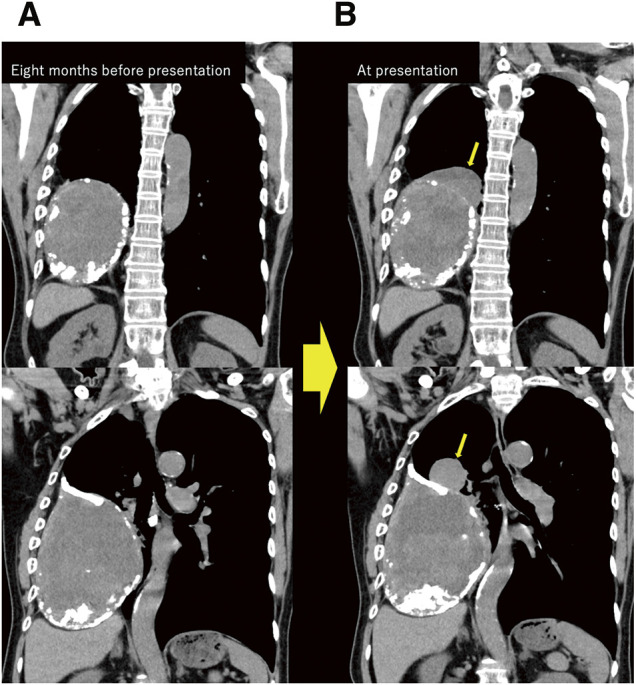
Comparison of coronal contrast-enhanced chest CT images in the equilibrium phase obtained 8 months before presentation (**A**) and at presentation (**B**), showing a mass in the right pleural cavity with peripheral calcification and enhancement of the lesion wall. 2 cranially projecting components developed during the interval (arrows).

Chest radiography (not shown) and computed tomography (CT) revealed a well-defined mass in the right thoracic cavity with peripheral calcifications and partial enhancement of the lesion walls, indicating ongoing neovascularization. In addition, 2 cranially projecting components were clearly observed on coronal contrast-enhanced CT. Serial imaging demonstrated progressive enlargement of the lesion over an 8-month period prior to presentation (**Fig. 1**). Magnetic resonance imaging revealed a heterogeneous high-intensity signal around the lesion on T2-weighted images. The heterogeneous signal of the lesion and a thin pseudocapsule with low signal intensity was observed on both T1- and T2-weighted images, consistent with a chronic hematoma (image not shown). Based on the above findings, CEH was strongly suspected, while the possibility of malignancy was considered low.

Bronchoscopy revealed occlusion of the right middle and lower lobes, with difficulty passing the bronchoscope. The source of bleeding was identified at the entrance of the right B7, B8, B9, and B10 bronchi. Tests for tumor markers and interleukin-2 yielded negative results. Based on these radiological and clinical findings, CEH was considered the most likely diagnosis.

### Treatment and outcome

Based on the results of a multidisciplinary conference involving thoracic surgery and respiratory medicine, and in consideration of the patient’s age and surgical risks, TAE was selected as the treatment strategy. Percutaneous biopsy was considered, but it was not performed due to the risk of bleeding.^2)^

**Fig. 2:**
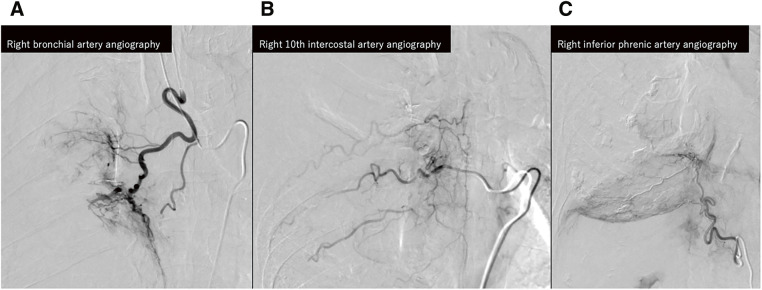
Angiographic identification of the feeding arteries to the hematoma. (**A**) Right bronchial artery supplying the cranial portion. (**B**) The right 10th intercostal artery, supplying the medial portion. (**C**) The right inferior phrenic artery, supplying the caudal portion. All identified feeding vessels were embolized using 700–900 μm Embosphere microspheres (Merit Medical Systems, South Jordan, UT, USA) and gelatin sponge particles (Serescue; Astellas Pharma, Tokyo, Japan).

TAE was conducted as follows (**Fig. 2**): A 5-Fr-long sheath was inserted via the right femoral artery, and the right bronchial artery was selected using a Mikaelsson catheter. Subsequently, a triple coaxial system (Meister S14 [Asahi Intecc, Aichi, Japan], Carry HF [UTM, Aichi, Japan], and Carry Selective [UTM]) was advanced into the bronchial artery, and digital subtraction angiography (DSA) was performed. No apparent arteriovenous shunt or spinal artery branching was observed. Embolization was performed using EmboSphere (Merit Medical Systems, South Jordan, UT, USA) and a gelatin sponge (Serescue; Astellas Pharma, Tokyo, Japan) through a Carry HF microcatheter. 1 vial of Embosphere microspheres was used. The microspheres were suspended by mixing 11 mL of buffering solution with 9 mL of contrast medium to obtain a 20-mL suspension. This mixture was divided into a 5-mL portion, to which an additional 15 mL of contrast medium was added to reach a final volume of 20 mL, and was administered under continuous agitation. Additional embolization with the gelatin sponge was performed once the abnormal enhancement of the hematoma disappeared. Next, the right 10th intercostal artery was selected, and abnormal staining was noted on angiography. Embolization was performed in the same way. The right inferior phrenic artery was then selected using a Shepherd hook catheter. Angiography demonstrated extensive abnormal staining along the caudal aspect of the large mass, and embolization was carried out. Post-embolization angiographic evaluation was performed to identify any residual feeding arteries. Selective angiography of the right subclavian artery using a Head Hunter catheter demonstrated no arterial supply to the hematoma. Subsequently, angiography of the ascending aorta was performed with a pigtail catheter, which also revealed no feeding vessels (not shown). A 7-Fr-long sheath was then introduced via the right femoral vein, and pulmonary angiography was conducted using an angio-Bermann catheter; however, no contrast blush or abnormal vascular supply was observed (not shown).

**Fig. 3:**
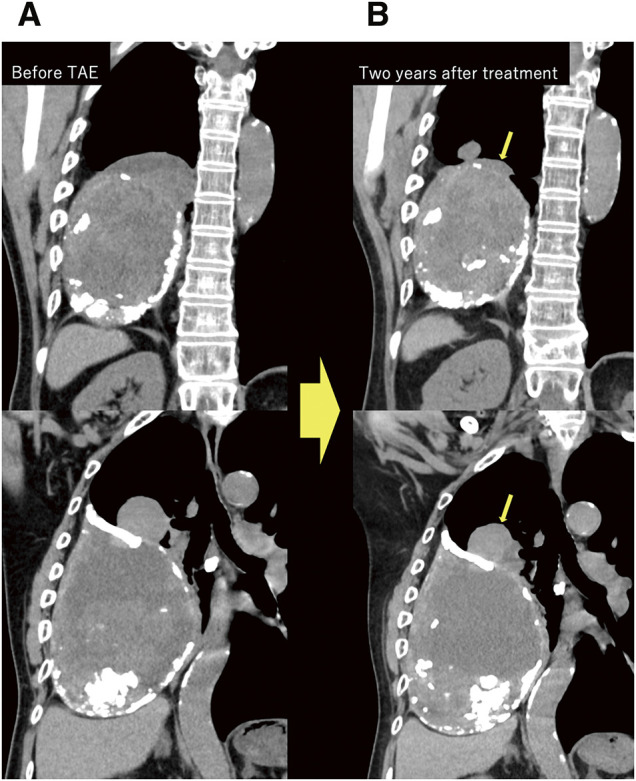
Coronal chest CT images before TAE (**A**) and 2 years after treatment (**B**). The cranial protrusions demonstrate significant size reduction at follow-up (arrows).

2 months after the procedure, follow-up chest radiography and CT revealed a reduction in hematoma size (**Fig. 3**). Further, the patient's symptoms, including hemoptysis, had completely resolved. No recurrence of symptoms or hematoma regrowth was observed during the 2-year follow-up period, and no major complications occurred.

## Discussion

CEH typically occurs long after an initial triggering event, such as trauma, surgery, or infection.^1)^ In the thoracic cavity, CEH generally occurs as a late complication of surgery or chest trauma. The patient’s history of artificial pneumothorax surgery for tuberculosis is considered to have contributed to the development of CEH.

The progressive nature of CEH of the thoracic cavity can mimic malignancies such as lymphomas, sarcomas, and pleural mesotheliomas in imaging studies.^4)^ Recognizing CEH is important to avoid any unnecessary aggressive interventions, particularly in patients with a history of thoracic surgery, trauma, or infection. In this case, although histopathological confirmation was not available, the diagnosis of CEH was supported by the imaging findings and clinical course.

The slow, progressive growth of the CEH lesion is driven by recurrent minor bleeding into the hematoma cavity, facilitated by fragile neovascularization, increased vascular permeability, and the formation of a fibrous capsule around blood products. Destructive products from blood cells within the thrombus trigger inflammation, leading to fibroblast formation and the development of a membrane and new vessels around the thrombus. As blood leaks from these fragile vessels and plasminogen activators increase permeability, the hematoma expands over time, potentially causing clinical symptoms, such as hemoptysis or compression of nearby structures.^5)^ Although complete surgical resection of the hematoma and its capsule is considered the gold-standard treatment, it can be highly invasive because CEH often adheres to surrounding structures, requiring extended resection and carrying a substantial risk of intraoperative bleeding and adjacent organ injury.^1)^ For this reason, preoperative TAE is performed to decrease vascularity and reduce surgical risk. When complete excision is technically difficult or contraindicated, TAE itself may serve as an alternative treatment option. With the appropriate selection of embolic materials, TAE can achieve effective devascularization, prevent further enlargement of the hematoma, and may contribute to gradual size reduction over time. In our patient, surgery was considered risky owing to advanced age and multiple comorbidities. Therefore, we chose TAE, which resulted in significant symptom resolution and stabilization of hematoma size over 2 years.

Previous reports have primarily used a gelatin sponge or metallic coils; however, the gelatin sponge provides only temporary occlusion, and coil embolization may result in proximal occlusion. For these reasons, we selected Embosphere as a permanent embolic agent in the present case, which contributed to achieving a durable therapeutic effect over the long term. Embolization using larger-sized tris-acryl microspheres (700–900 μm) may reduce the risk of complications.^6)^ Because particles smaller than 325 μm may migrate through bronchopulmonary shunts and cause unexpected non-target embolization,^7)^ we selected larger Embosphere particles in this case. Smaller particles may also inadvertently migrate into spinal arterial feeders and other non-target branches. Accordingly, larger Embosphere particles (700–900 μm) were selected in this case to minimize the risk of distal non-target embolization.

Other studies have previously reported that bronchial artery embolization with 700–900 μm Embosphere particles is a safe and effective treatment for massive hemoptysis.^8)^ In our case, no major complications were observed, and favorable therapeutic outcomes were achieved, indicating that this technique with 700–900 μm Embosphere particles may be a safe and useful option for treating CEH.

Recent studies have shown similarities between CEH and chronic subdural hematoma in terms of histopathological and biochemical characteristics.^9)^ Both conditions involve chronic bleeding and the formation of a fibrous capsule surrounding the hematoma. Given these similarities, treatment strategies that have proven effective for chronic subdural hematoma may also be applicable to the management of CEH. For example, middle meningeal artery embolization is a promising minimally invasive therapy that can reduce the need for surgical intervention in appropriately selected patients with chronic subdural hematoma.^10)^ This technique can also effectively reduce recurrence rates and stabilize the hematoma by interrupting its blood supply. For chronic subdural hematoma (CSH), there have been reports of using particles sized 100–300 and 300–500 μm for middle meningeal artery embolization (MMAE).^11)^ Similarly, TAE may prevent massive bleeding from the hematoma wall in patients with CEH. Preoperative embolization can also play a crucial role in managing patients in whom complete surgical excision is either too risky or technically unfeasible. In such cases, embolization reduces the risk of intraoperative hemorrhage. Only one previous report described a case in which the hematoma decreased in size following arterial embolization alone; however, the type of embolic material used was not specified.^2)^ In addition, we found no other studies demonstrating the efficacy of TAE as a standalone treatment for CEH.

Based on the results of this case, TAE is considered a viable treatment option for patients with thoracoabdominal CEH in whom complete surgical resection is challenging or surgery is not feasible due to poor general conditions.

This case report has several limitations. First, the diagnosis of CEH was made based on clinical and imaging findings without histopathological confirmation. Second, long-term follow-up beyond 2 years was not available, limiting the assessment of delayed complications or recurrence.

## Conclusion

Overall, the present case suggests that TAE may be an effective treatment option for CEH in the thoracic cavity, particularly in cases in which surgical resection is not feasible because of comorbidities or high surgical risk. Overall, this case demonstrates the successful use of TAE to control symptoms and stabilize lesions over a long follow-up period.
